# Effect of root-extracts of *Ficus benghalensis* (Banyan) in memory, anxiety, muscle co-ordination and seizure in animal models

**DOI:** 10.1186/s12906-016-1413-5

**Published:** 2016-11-03

**Authors:** Dipesh Raj Panday, G. P. Rauniar

**Affiliations:** Department of Clinical Pharmacology & Therapeutics, B.P. Koirala Institute of Health Sciences (BPKIHS), Dharan, Nepal

**Keywords:** Ficus benghalensis L, Nepal, Neuropharmacological parameters, Open-field test, Passive-avoidance test, Pentobarbital-induced Sleep potentiation test, Pentylenetetrazol-Induced Seizure Test. Rota-rod test

## Abstract

**Background:**

Ficus benghalensis L. (Banyan) is a commonly found tree in Eastern Nepal. Its different plant parts are used for various neurological ailments. This study was performed in mice to see its effects in various neuropharmacological parameters.

**Methods:**

Passive-avoidance (memory), Open-field (anxiety), Pentobarbital-induced Sleep potentiation (sleep), Rota-rod (muscle-co-ordination), Pentylenetetrazol-Induced and Maximal Electroshock Seizure Tests were performed. Sample size was calculated using G*Power 3.1.9.2. Aqueous root extracts (Soxhlet method) of Ficus benghalensis 100 mg/kg and 200 mg/kg with negative and positive controls were used. The experimental results were represented as Mean ± SD. P-value was set at <0.05. Oneway analysis of variance (ANOVA) or Mann-Whitney *U* test was appropriately used.

**Results:**

Passive-avoidance test showed 200 mg/kg group spent significantly less. Time (0.00s + 0.00s) in shock-zone than Normal Saline-group (9.67 s + 14.36 s, *P* = 0.000) or Diazepam-group (41.07 s + 88.24 s, *P* = 0.000).

Open-field test showed 200 mg/kg group spent significantly longer Time (24.77 s + 12.23 s) in central-square than either Normal Saline group (15.08 s + 6.81 s, *P* = 0.000) or Diazepam-group (15.32 s + 5.12 s, *P* = 0.000).

In Rota-rod test, 200 mg/kg group fell off the rod significantly (*P* = 0.000) earlier (33.01 s + 43.61 s) than both Normal Saline (>120 s) and Diazepam (62.07 s + 43.83 s) PTZ model showed that 100 mg/kg significantly (*P* = 0.004) delayed seizure-onset (184.40s + 36.36 s) compared to Normal Saline (101.79 s + 22.81 s), however, in MES model 200 mg/kg significantly (*P* = 0.000) prolonged tonic hind-limb extension (17.57 s + 2.15 s) compared to Normal Saline (13.55 s + 2.75 s) or Phenytoin (00.00s + 00.00s).

**Conclusion:**

Aerial roots of Ficus benghalensis have memory-enhancing, anxiolytic, musclerelaxant, and seizure-modifying effect.

**Electronic supplementary material:**

The online version of this article (doi:10.1186/s12906-016-1413-5) contains supplementary material, which is available to authorized users.

## Background

According to the WHO (World Health Organization), 70–80 % of the population in developing countries still relies on non-conventional medicine mainly of herbal sources in their primary healthcare. Even in allopathy, pharmacological screening of plants often provide basis for developing new lead molecules [1, 2]. The recent advancements in phyto-chemistry and molecular biology have renewed and rejuvenated our interest in herbal medicines. Again, anecdotal claims of ‘no side effects’, easy access and cheap price have lured people from all walks of life to turn back to nature [3].

Nepal, a repository of wild flora and fauna, harbors 2.2 % of world’s flowering plants in spite of sharing only 0.1 % of the total land area of the world. Out of the 7000 species of higher plants available in Nepal, recent literature claims that, more than 1400 are of medicinal value [4–6].

*Ficus benghalensis* L., according to International Plant Names Index (IPNI), is ‘Bar’ in Nepali, ‘Banyan’ in Hindi and ‘East Indian fig tree’ or ‘Indian Banyan’ in English. It is taxonomically sub-classified under Order- Rosales; Family- Moraceae; Genus-*Ficus* and Species- *benghalensis* [7].

In traditional system of medicine, various plant parts of *Ficus benghalensis* L.such as stem bark, aerial roots, underground roots, vegetative buds, leaves, fruits and latex have been used in various nervous disorders i.e. seizure, insomnia, anxiety etc. [8, 9]. Basu and Lal described many neuropharmacological effects of the plant [10].

Several of its pharmacological activities have also been scrutinized under scientific light. In Passive-avoidance test and plus-maze tests on scopolamine-induced amnesia in young mice, Chandra et al. [11] found that aqueous extracts of Ficus benghalensis L.bark did have cognitive enhancing activity.

Deraniyagaia [12] however could not verify significant sedative effects of aqueous leaf extract of *Ficus benghalensis* L.

There have been many attempts to prove analgesic and anti-inflammatory [2, 13], antistress and antiallergic [14], antimicrobial [15, 16], antiplasmodial [17], mosquito larvicidal [18] anti-ulcer [19], antifungal [20], anti-lipidemic [21] anti-atherogenic [22] antidiabetic [23, 24] properties of various parts of the plant. Though mainstream medicine has claimed several interesting findings, no break-through has yet been achieved [25].

It is shocking to find very scarce scientific studies especially from our country trying to verify our local traditional claims and uses. Few scientific studies done elsewhere may not be the answer to our different climatic and social perspectives. We, therefore, undertook this study to scrutinize whether the aqueous extracts of aerial roots of *Ficus benghalensis* L.do have any effect in memory, anxiety, sleep, muscle co-ordination, and seizure in animal-models.

## Methods

### Setting

Laboratory of Department of Clinical Pharmacology and Therapeutics, BPKIHS, Dharan, Eastern Developmental Region, Nepal.

Experiments were performed between 8:00 and 16:00 h.

Experiments were conducted 2014 March-May when the average temperature was between 11 to 21 ˚C.

### Sample, sampling technique and animals

Sample size was calculated using sample-size calculating software G*Power version 3.1.9.2 (Program written, concept and design by Franz, Universitat Kiel, Germany. Freely available windows application software). With power of 80 %, 0.05 statistical level of significance and effect size of 0.8, sample size for each test was calculated to be 24. Therefore, for six different tests, sample size was 6 × 24 = 144.

One hundred and fourty four Swiss albino mice were randomly assigned into one of the four experimental groups.

The mice were produced in the breeding house of Department of Clinical Pharmacology and Therapeutics, BPKIHS, Nepal.

The mice had free access to food and water and were fasted for 12 h before the experiments.

### Design of the study

It was a Quantitative experimental study in mice.

### Eligibility

#### Inclusion criteria

- Swiss albino mice of either sex.
- Weighing 20–30 g.

#### Exclusion criteria

Apparently, free of any disease or handicap.

### Study flow chart (Fig. 1)

**Fig. 2:**
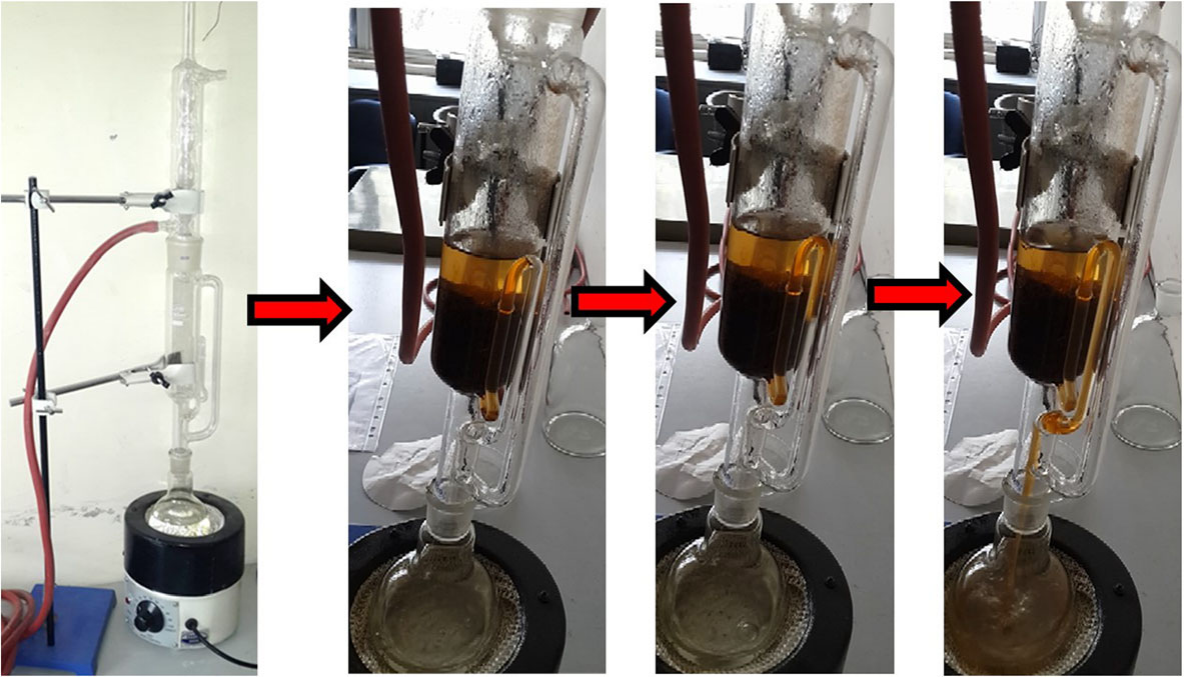
Soxhlet apparatus in use

### Test drug preparation (Retention sample number: DDRP-1) (Fig. 2)

A botanist authenticated a *Ficus benghalensis* L. *tree* in the premise of BPKIHS, eastern part of Nepal, with Latitude and Longitude 26° 49′ 0″ N and 87° 17′ 0″ E respectively. Sample specimen with Retention sample number DDRP-1, was deposited to National Herbarium & Plant Laboratories, Nepal under Department of Plant Resources, Thapathali, Kathmandu. In February 2014, about 2.5 kg aerial roots of the tree was carefully collected, thoroughly washed with tap*-*water, shade-dried for several days and pulverized to fine powder in a mixer (Panasonic MX AC 400 Mixer Grinder, 550 W Power Consumption, Jaipur, India). About 2 kg of resulting crude root-powder was extracted in several batches using soxhlet apparatus (JAIN SCIENTIFIC GLASS WORKS AMBALA CANTT; Extraction Pot: 250 ml; Soxhlet chamber size: 100 ml; Heater: DICA India). About 250 ml of Distilled water was used in each batch of extraction. Each batch was extracted for 24 h. Thus produced aqueous root extract was heated in 50̊ C for a brief time interval, stopped just before the apparently saturated solution precipitated and left in room temperature till the moisture dried. Two kg crude root powder yielded 102.68 g extract (5.13 %) by soxhlet method [26]. Thus resulted dried powder extract was safely stored in a dry air-tight plastic container till the day of experiment [27]. On the experiment-day, 20 mg/ml and 10 mg/ml solutions in distilled water were prepared by serial dilution such that 1 ml/100 g mouse body weight could be injected to the mice in test-drug group for the desired test dose of 200 mg/kg and 100 mg/kg respectively. On the day of experiment, the previous-day solutions were discarded and fresh solutions prepared.

**Fig. 3:**
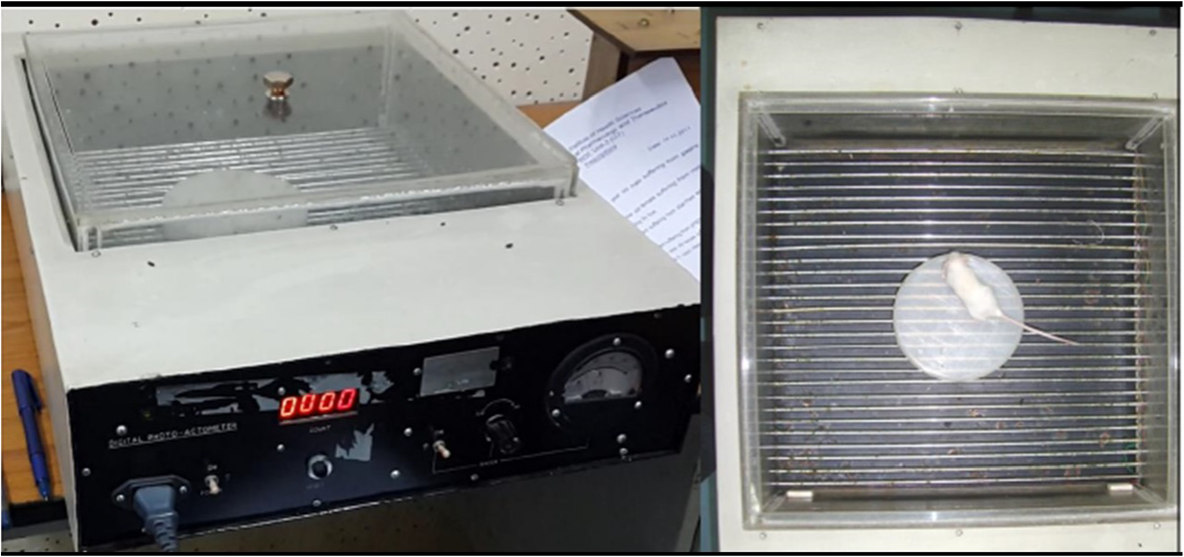
Passive-avoidance test apparatus

### Timing of the drug administration

All paroral (PO) drugs were carefully administered with the help of oro-gastric tube 60 min prior to the intended test. All parenteral drugs were given Intrperitioneal (IP) [28–30].

### Drugs and chemicals used in experimental models

1. Test drug -Aqueous solutions of aerial root extracts of *Ficus benghalensis* L.100 mg/kg and 200 mg/kg were given PO
2. Negative control in all 6 models: Normal Saline (Claris Life Sciences) was given PO
3. Positive control
  - MES seizure → Phenytoin (M-Toin, Medopharm, India) 20 mg/kg PO [30]
  - Rota-rod test: Diazepam (Valium, Piramal Healthcare, India) 4 mg/kg IP [28]
  - PTZ (Pentylenetetrazole) test: Diazepam 4 mg/kg IP [31]
  - Open-field test: Diazepam 1 mg/kg IP [30]
  - Passive-avoidance test: Diazepam 1 mg/kg IP [30]
  - Pentobarbital induced sleep potentiation test: Diazepam 3 mg/kg IP [30]
4. Inducing agents
5. Sleep in Pentobarbital-induced sleep potentiation test: Pentobarbital (LobaChemie, India) IP 50 mg/kg [30]
6. Seizure in PTZ seizure: Pentylenetetrazol (PTZ) (Sigma Chemicals, USA) 50 mg/kg IP [30]

### Passive-avoidance test (Fig. 3)

In a dark, sound-attenuated experimental room was the study chamber for Passive-avoidance Test, which consisted of grid metal floor of 34 × 34 × 20 cm dimension. In the metal floor, 20 mA was being run. A shock free zone (SFZ) was created at the center by placing an inverted Petri-dish.

**Fig. 4:**
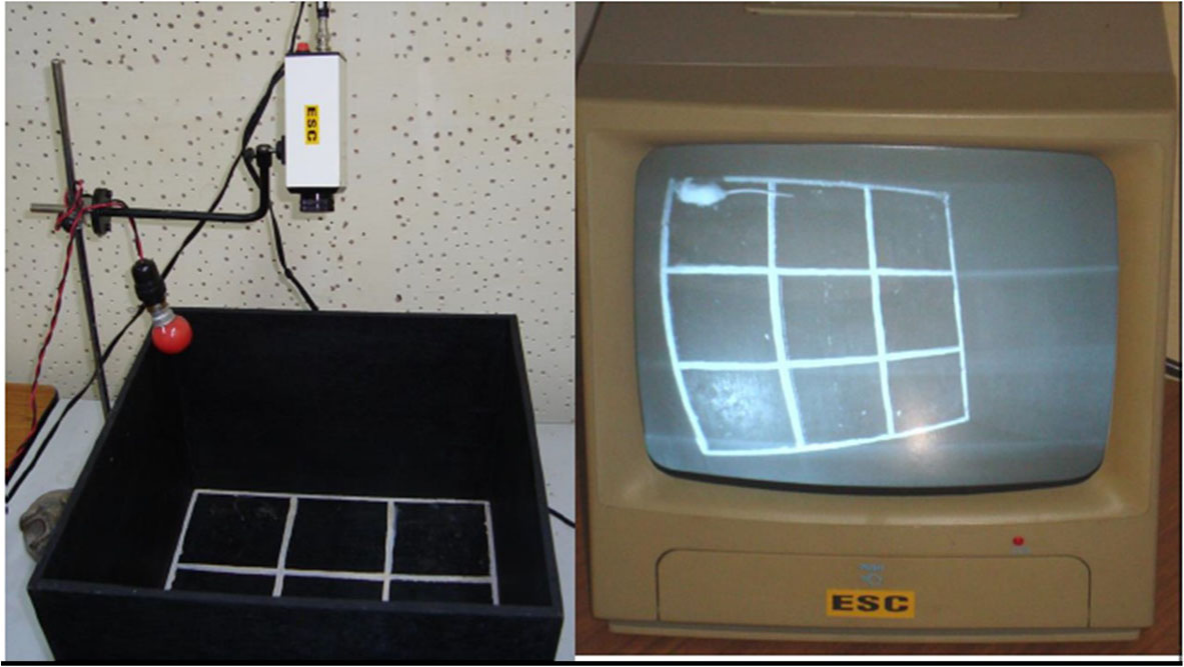
Open field test apparatus set-up

Prior to the actual documentation of the parameters every mouse underwent a training session in which each was left on the live metal grid for sometimes till it became apparent that it had identified shock-zone (Grid-metal floor) and shock-free zone (Petri-dish) where it would be safe from electric shock. This was the principle of avoidance. Those mice, who did not learn this avoidance technique in 5 consecutive training sessions were discarded. Learning was assumed when the mouse stayed on the petri-dish for at least 60s. Then, a learnt mouse who took oral drug 1 h back was placed on the Petri-dish and following parameters were documented for 5 consecutive minutes [30, 32].

1. Step-down latency (Initial duration for which the animal remained in SFZ till it moved to the shock zone).
2. Step-down error (Number of unsuccessful attempts made to go to the shock zone).
3. Total time spent in the shock zone.

An increase in Step- down latency and decrease in either step-down error or time spent in the shock zone indicated learning and memory [30, 32].

Diazepam 1 mg/kg IP [30], the positive control of the test, was administered 15 min prior to the test.

### Open field test (Fig. 4)

In a dark, sound-attenuated experimental room was the open-field apparatus vaguely illuminated with a zero Watt red-bulb. Open Field consisted of a wooden box (40 cm × 40 cm with 30 cm high walls) with black floor, subdivided into nine (3 vertical X 3 horizontal) equal squares, by 4 (2 horizontal and 2 vertical) white lines. One hour after the oral drug administration, the mouse was placed in the open field. There, it was left undisturbed for about 1 min so that it could acclimatize to this new surroundings. Then, following parameters were documented in the next 5 min [33].

- Ambulation (number of square crossed)
- Time spent in the central square
- Rearing (number of times the animal standing on the rear paws)

are recorded.

**Fig. 5:**
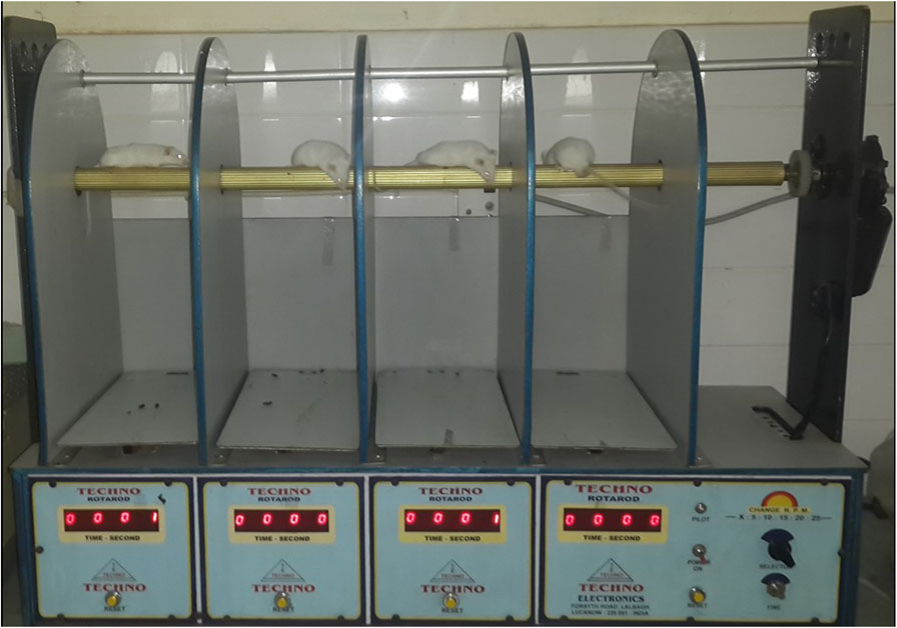
A Rota-rod Treadmill device

In an anxious situation, a rodent tends to reduce exploratory activities like ambulation, rearing (an animal standing on the rear paws) and shows spontaneous preference to the periphery of the apparatus. Anxiety decreases all three parameters [33].

Diazepam 1 mg/kg IP [30], the positive control of the test, was administered 15 min prior to the test.

### Pentobarbital-induced sleep potentiation test

One hour after the oral drug administration, every mouse received Pentobarbital 50 mg/kg IP [34] The time that elapsed between loss and recovery of righting reflex was taken as the sleeping time. The mouse was considered as being awake if it could right itself, i.e. return to upright position. Diazepam 3 mg/kg IP [30], the positive control of the test, was administered, 15 min prior to the Pentobarbital administration.

### Rota-rod test (Fig. 5)

A Rota-rod Treadmill device (Techno, India) was used. One hour after the oral administration of the drug, the mouse was placed on a horizontal rotating rod revolving at 15 revolution per minute [35]. Before that, mice were trained to adjust to the revolving rod. Only those mice which demonstrated their ability to remain on the revolving rod for at least 1 min were used for the test [30]. ‘Fall-off time’ when the mouse fell from the rotating rod was noted. For mice, who did not fall, observation was made till 2 complete minutes [31]. Diazepam 4 mg/kg IP [28], the positive control of the test, was administered 15 min prior to the test.

**Fig. 6:**
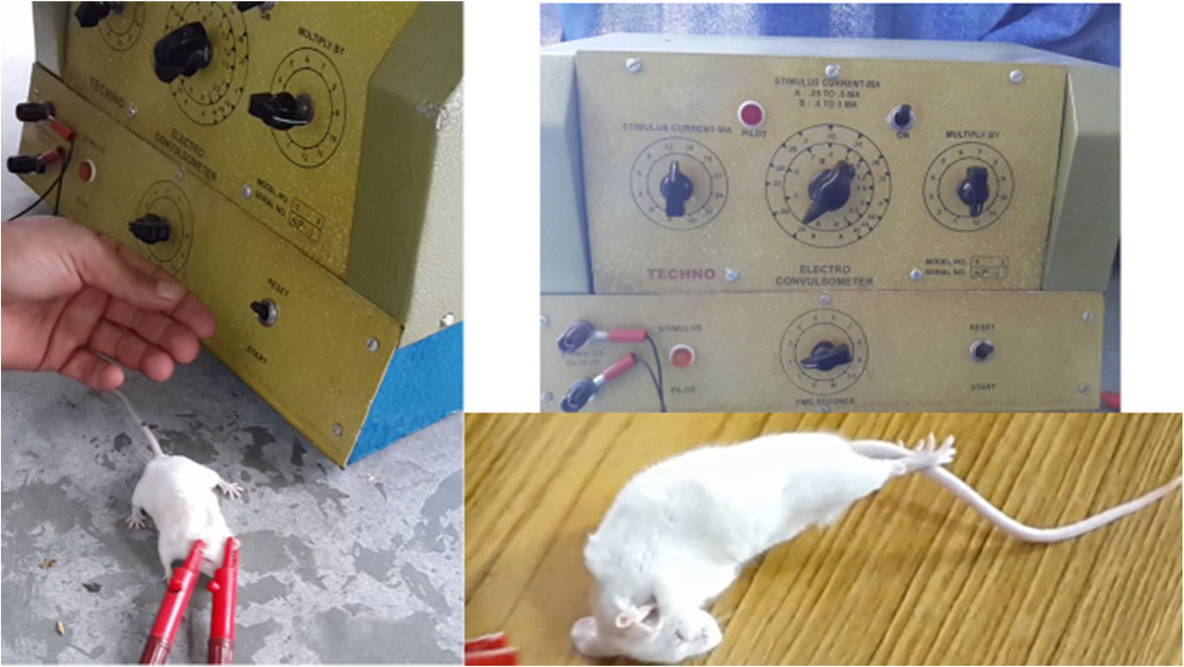
Convulsiometer and Tonic hind limb extension seen in MES test

### Anticonvulsant effect

#### Pentylenetetrazol (PTZ) induced seizure test

One hour after the oral drug administration, 60 mg/kg [30] PTZ IP (Intraperitoneal) was administered to all mice. The documented parameters were latency to seizure-onset and the number of mice not exhibiting seizure. Seizure was defined as jerky movements of whole body or convulsion [28]. Each mouse was observed for one whole hour for the occurrence of seizure. Diazepam 4 mg/kg [31] IP, the positive control of the test, was administered 15 min prior to the test.

#### Maximal Electroshock (MES) Seizure Test (Fig. 6)

One hour after the oral drug administration, the mouse was subjected to alternating current of 150 mA, 50 Hz for 0.2 s duration. Current was produced by Convulsiometer (Techno, India) and was applied through a pair of electrodes attached to either ear [36]. Each animal was observed for 2 complete minutes [30]. Parameters observed and documented were

- Duration of tonic hind limb extension.
- Number of animals not showing seizure (animals protected against seizure).

Phenytoin 20 mg/kg PO [30] was the positive control of the test.

**Fig. 7:**
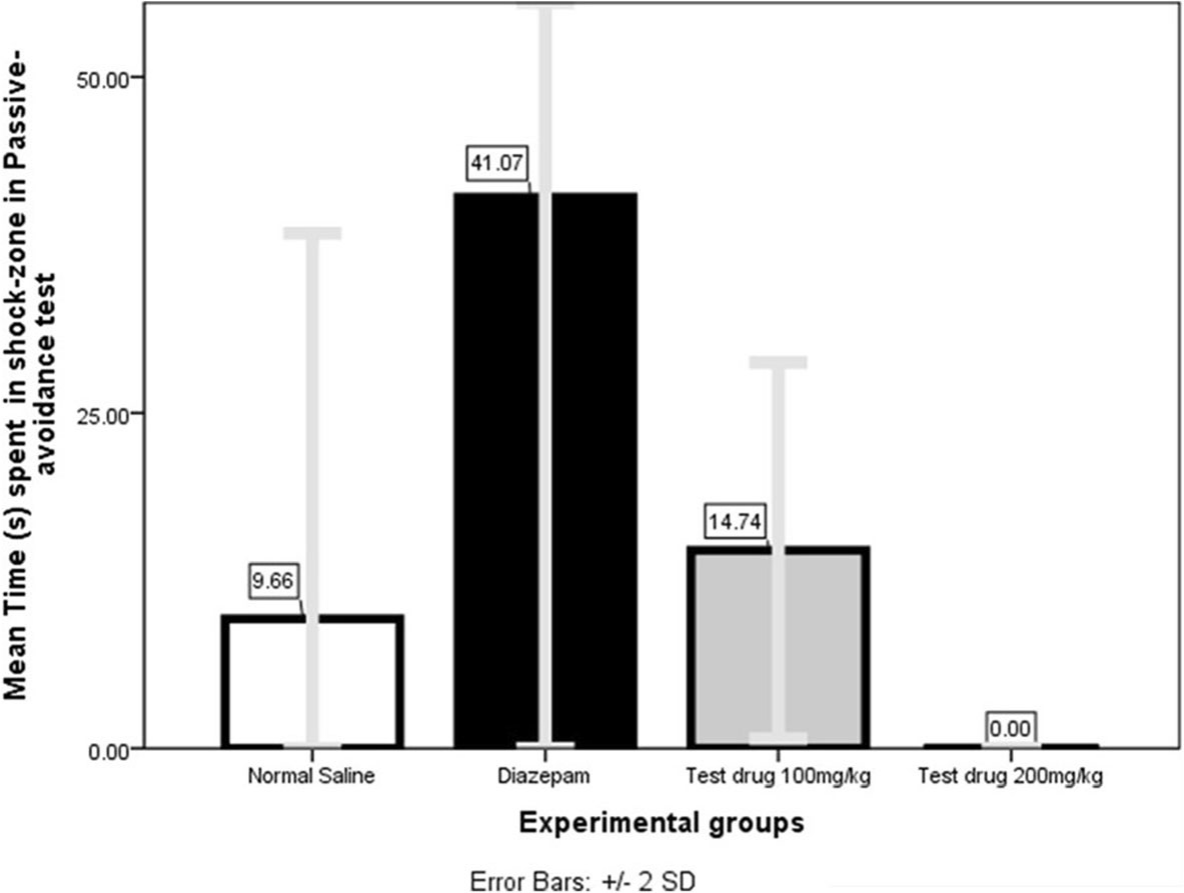
Comparison of Mean Time spent in shock-zone (s) in Passive-avoidance test among 4 different experimental groups

### Statistical analysis

The experimental results were represented as Mean ± SD (Standard Deviation). SPSS (Statistical Package for the Social Sciences) version 21 {Property of International Business Machines (IBM) Corporation, SPSS is the registered trademarks of the corporation (IBM)} was used. For normally distributed dependent variable, parametric-test-one way analysis of variance (ANOVA) followed by Tukey’s multiple comparison was applied. For those, not normally distributed, non-parametric test-Mann–Whitney *U* test was employed for comparison. Statistical level of significance was set at *P-*value < 0.05.

### Ethical considerations

Prior to the conduction of the experiment, ethical clearance was obtained from the Ethical Committee on Animal Research of BPKIHS. Maximum precaution was taken to reduce the pain and injury to the animals in the course of experiment yet not compromising the standard of the experiment. Appropriate help was procured from expert experimental animal handlers during the course of study.

## Results

### Passive-avoidance test (Table 1)

#### Step-down latency (s)

One‑way ANOVA (Additional file 1: Table S3) revealed a significant *(P =* 0.001) result on mean Step-down latency. Multiple Comparisons (Additional file 1: Table S4) by Tukey HSD post hoc tests (α = 0.05) showed only 200 mg/kg group had a significantly *(P =* 0.014) higher mean Step-down latency (300.00 s ± 0.00s) compared to Diazepam-group (121.17 s ± 114.22 s).

**Table 1:** Comparison of parameter (s) in Passive-avoidance test among 4 different experimental groups

Experimental group	Mean Step down latency ± SD (s)	Mean Step down errors ± SD	Mean Time spent in shock zone ± SD (s)
Negative Control (Normal Saline)	171.00 ± 142.21	15.17 ± 8.28	9.67 ± 14.36
Positive Control (Diazepam)	121.17 ± 114.22	6.67 ± 6.98	41.07 ± 88.24
Test drug 100 mg/kg	38.39 ± 9.59	23.17 ± 4.67	14.74 ± 7.01
Test drug 200 mg/kg	300.00 ± 0.00	8.83 ± 4.79	0.00 ± 0.00

#### Step down error in passive- avoidance test

One‑way ANOVA (Additional file 1: Table S7) revealed a significant *(P =* 0.001) influence on mean Step-down error. Multiple Comparisons (Additional file 1: Table S8) by Tukey HSD (Honest Significant Difference) post hoc tests (α = 0.05) showed that 100 mg/kg group produced significantly *(P =* 0.001) higher mean Step-down error (23.17 s ± 4.67 s) compared to Diazepam-group (6.67 s ± 6.98 s).

#### Mean Time spent in shock-zone (s) (Fig. 7)

Mann–Whitney U (Additional file 1: Table S11) showed 200 mg/kg group spent less mean Time (0.00s ± 0.00s) in shock-zone which was significant compared both to Normal Saline-group (9.67 s ± 14.36 s, *P* = 0.000) and Diazepam-group (41.07 s ± 88.24 s, *P* = 0.000).

**Fig. 8:**
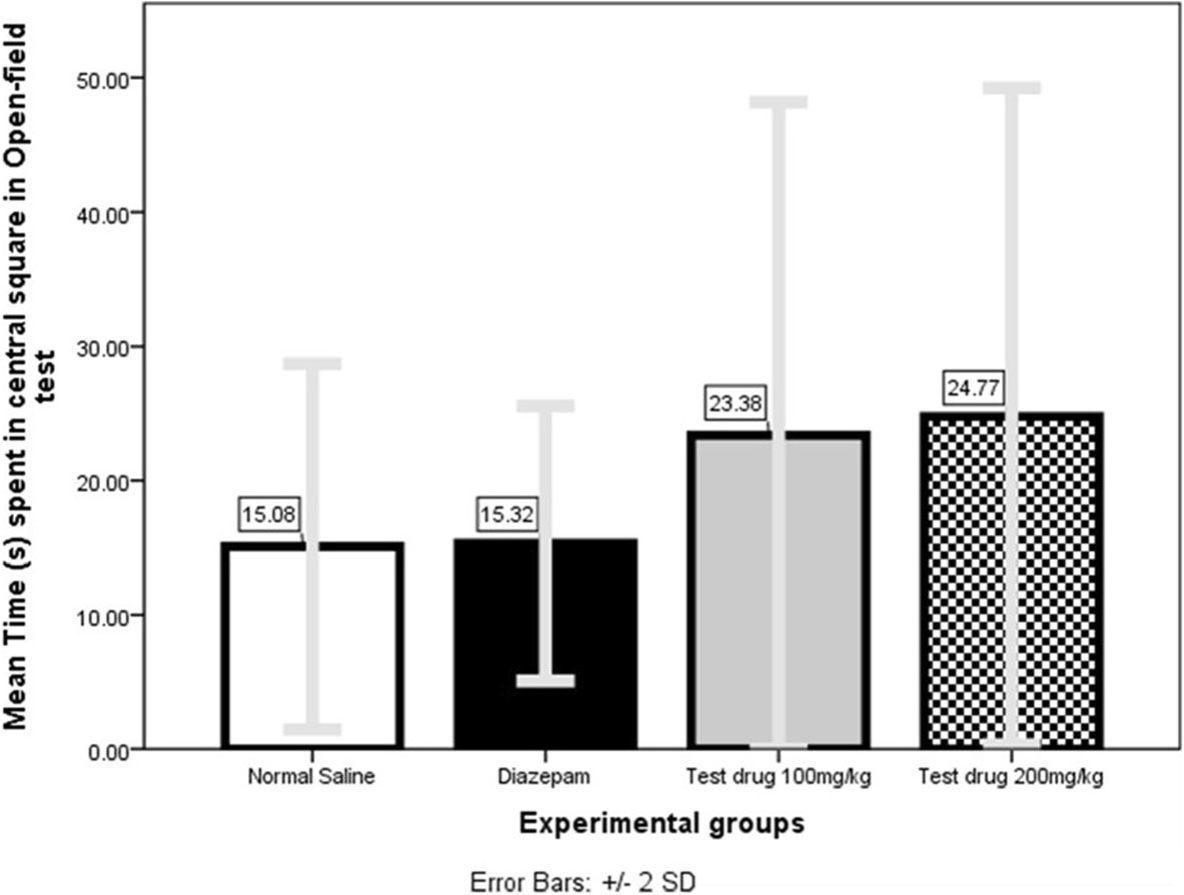
Comparison of Mean Time (s) spent in central-square in Open-field test among 4 different experimental groups

### Open-field test (Table 2)

#### Number of squares crossed

One‑way ANOVA (Additional file 1: Table S14) revealed a significant *(P =* 0.000) effect on mean number of squares crossed in open field test. Multiple Comparisons (Additional file 1: Table S15) by Tukey HSD post hoc tests (α = 0.05) showed mean number of squares crossed by group receiving both 100 mg/kg (184.17 ± 8.18,) and 200 mg/kg (123.33 ± 38.53,) were significantly *(P*_*=*_0.043, *P* = 0.00 respectively) lower than Diazepam-group (272.17 s ± 83.05 s).

**Table 2:** Comparison of parameters in Open-field test among 4 different experimental groups

Experimental Groups	Mean Number of squares crossed ± SD	Mean Time spent in central square ± SD (s)	Mean Number of rearing ± SD
Negative Control (Normal Saline)	131.33 ± 45.91	15.08 ± 6.81	41.00 ± 12.84
Positive Control (Diazepam)	272.17 ± 83.05	15.32 ± 5.12	45.83 ± 11.79
Test drug 100 mg/kg	184.17 ± 8.18	23.38 ± 12.39	51.67 ± 11.55
Test drug 200 mg/kg	123.33 ± 38.53	24.77 ± 12.23	36.17 ± 13.11

#### Mean time (s) spent in central-square (Fig. 8)

Mann–Whitney *U* test (Additional file 1: Table S18) showed 200 mg/kg group spent significantly longer Mean Time (24.77 s ± 12.23 s) in central-square when compared to either Normal Saline group (15.08 s ± 6.81 s, *P*_=_0.000) or Diazepam-group (15.32 s ± 5.12 s, *P*_=_0.000).

**Fig. 9:**
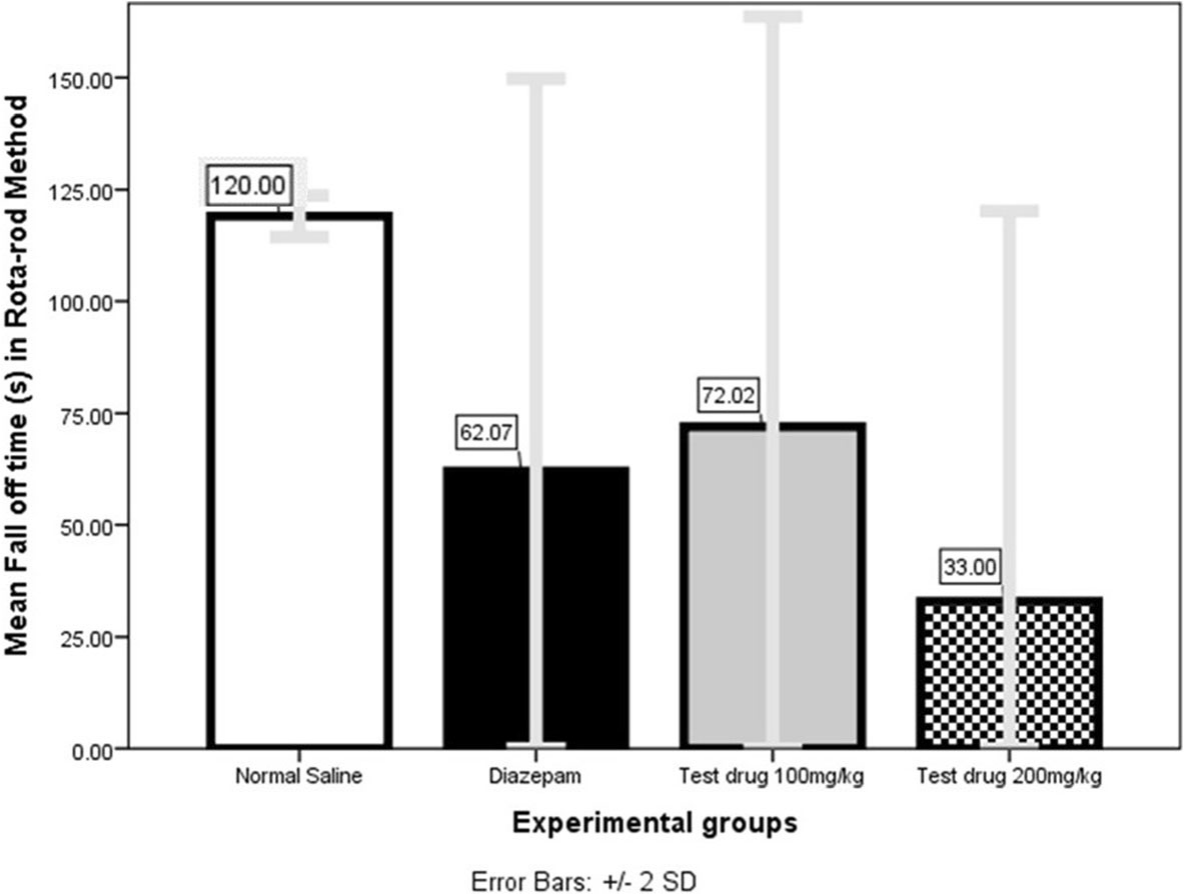
Comparison of Mean Fall-off time (s) in Rota-rod test among 4 different experimental-groups

#### Mean number of rearing

Mann–Whitney *U* test (Additional file 1: Table S21) showed non-significant (*p* > 0.05) influence of aqueous extracts of aerial roots of *Ficus benghalensis* L.on mean number of rearing among Swiss-albino mice.

### Pentobarbital-induced sleep potentiation test (Table 3)

One way ANOVA (Additional file 1: Table S24) revealed a significant *(P =* 0.00) effect on mean total sleep time. Multiple Comparisons (Additional file 1: Table S25) by Tukey HSD post hoc tests (α = 0.05) showed that both 100 mg/kg and 200 mg/kg groups slept significantly lesser mean duration (4796.50s ± 870.67 s, *P*_=_0.000, 4528.00s ± 1004.83 s, *P*_=_0.000 respectively) compared to Diazepam group (13707.33 s ± 5155.47 s).

**Table 3:** Comparison of Mean Total sleep time among 4 different experimental-groups

Experimental Groups	Mean Total sleep time ± SD (s)
Negative Control (Normal Saline)	6844.33 ± 545.95
Positive Control (Diazepam)	13707.33 ± 5155.47
Test drug 100 mg/kg	4796.50 ± 870.67
Test drug 200 mg/kg	4528.00 ± 1004.83

### Rota-rod test (Fig. 9 and Table 4)

Mann–Whitney *U* test (Additional file 1: Table S28) showed that at 100 mg/kg and 200 mg/kg the mean fall off time (72.02 s ± 45.82 s and 33.01 s ± 43.61 s respectively) were significantly lesser (both *P* = 0.000) than Normal Saline (>120 s). Again 200 mg/kg had significantly (*P* = 0.000) lower mean fall-off time (33.01 s ± 43.61 s) even when compared to Diazepam (62.07 s ± 43.83 s).

**Fig. 10:**
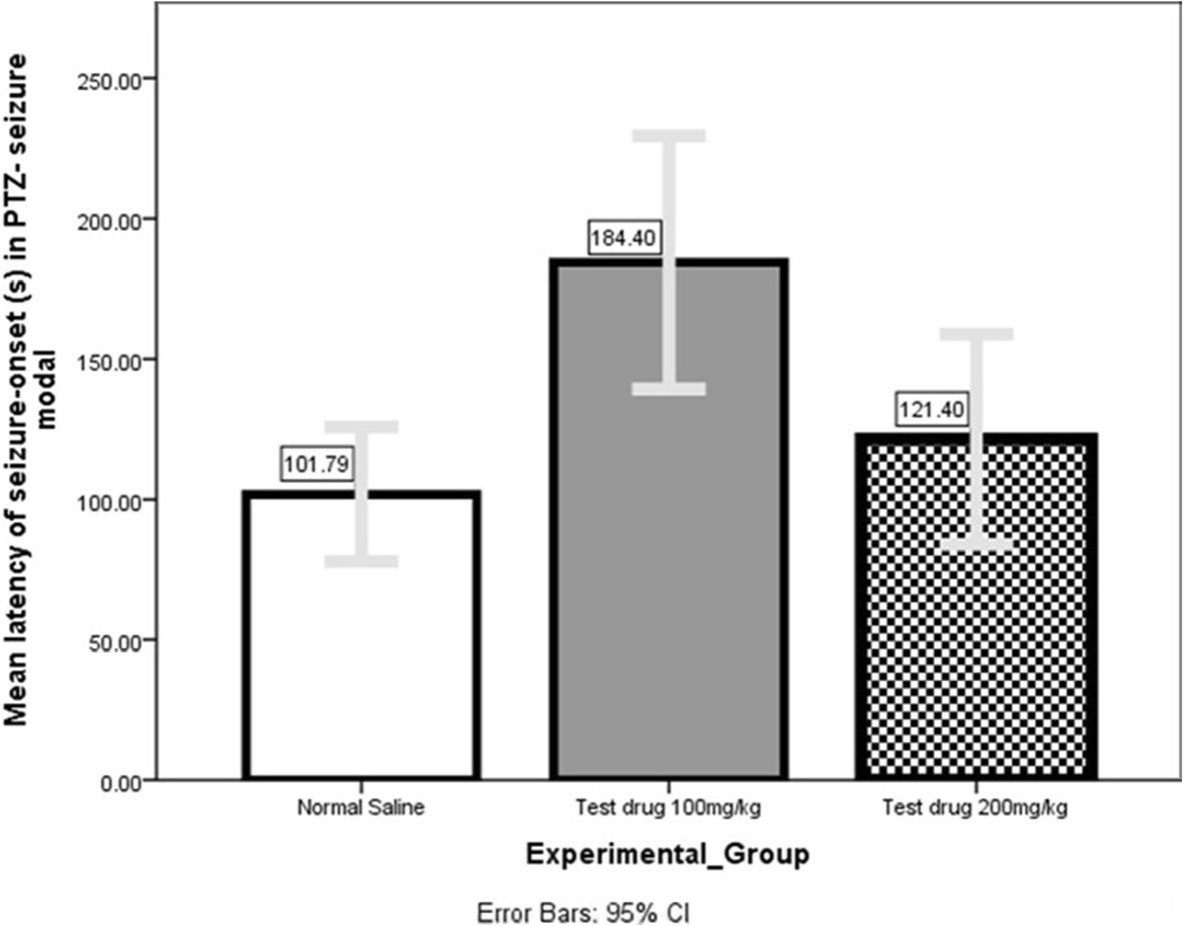
Comparison of Mean Latency of seizure-onset (s) in PTZ-seizure model among 3 experimental-groups showing seizure

**Table 4:** Comparison of Mean Fall-off time in Rota-rod test among 4 different experimental-

Experimental Groups	Mean Fall-off time ± SD (s)
Negative Control (Normal Saline)	More than 120
Positive Control (Diazepam)	62.07 ± 43.83
Test drug 100 mg/kg	72.02 ± 45.82
Test drug 200 mg/kg	33.01 ± 43.61

### PTZ test (Fig. 10 and Tables 5 and 6)

No animal died in PTZ seizure model.

Test drug at either dose protected 16.67 % (2/12 = 1/6 + 1/6) animals from seizure.

However, Mann–Whitney U (Additional file 1: Table S32) showed only 100 mg/kg could significantly *(P =* 0.004) delay seizure-onset (184.40s + 36.36 s) compared to Normal Saline (101.79 s + 22.81 s).

**Fig. 11:**
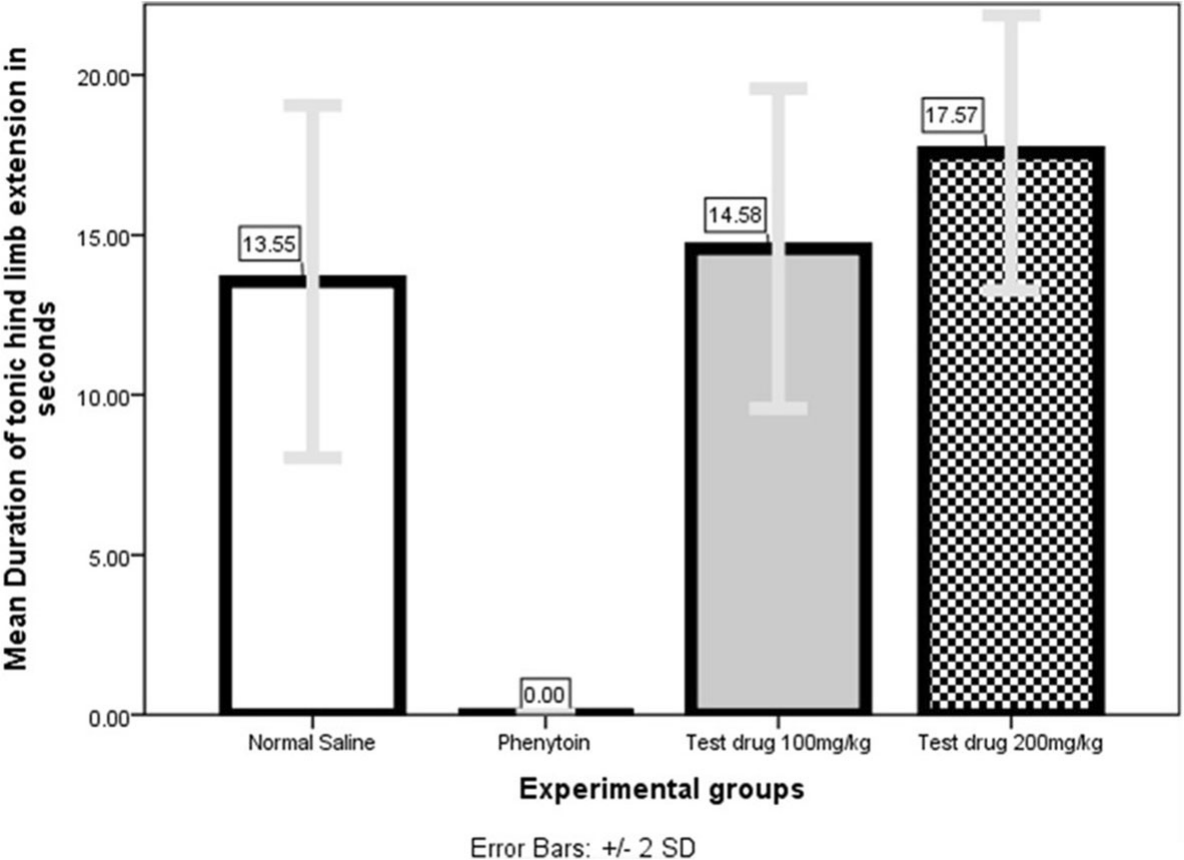
Comparison of Mean duration of tonic hind-limb extension in MES-seizure model among 4 different experimental-groups

**Table 5:** Animals not showing seizure in PTZ model

Experimental Group	Animals protected
Negative Control (Normal Saline)	0/6
Positive Control (Diazepam)	6/6
Test drug 100 mg/kg	1/6
Test drug 200 mg/kg	1/6

**Table 6:** Latency of seizure onset (s) in PTZ seizure model

Experimental Groups	Mean Latency of seizure onset ± SD (s)
Negative Control (Normal Saline)	101.79 ± 22.81
Positive Control (Diazepam)	No seizure seen in 1 h
Test drug 100 mg/kg	184.40 + 36.36
Test drug 200 mg/kg	121.40 + 30.15

### MES test (Fig. 11 and Tables 7 and 8)

No animal died in MES seizure model.

**Fig. 1:**
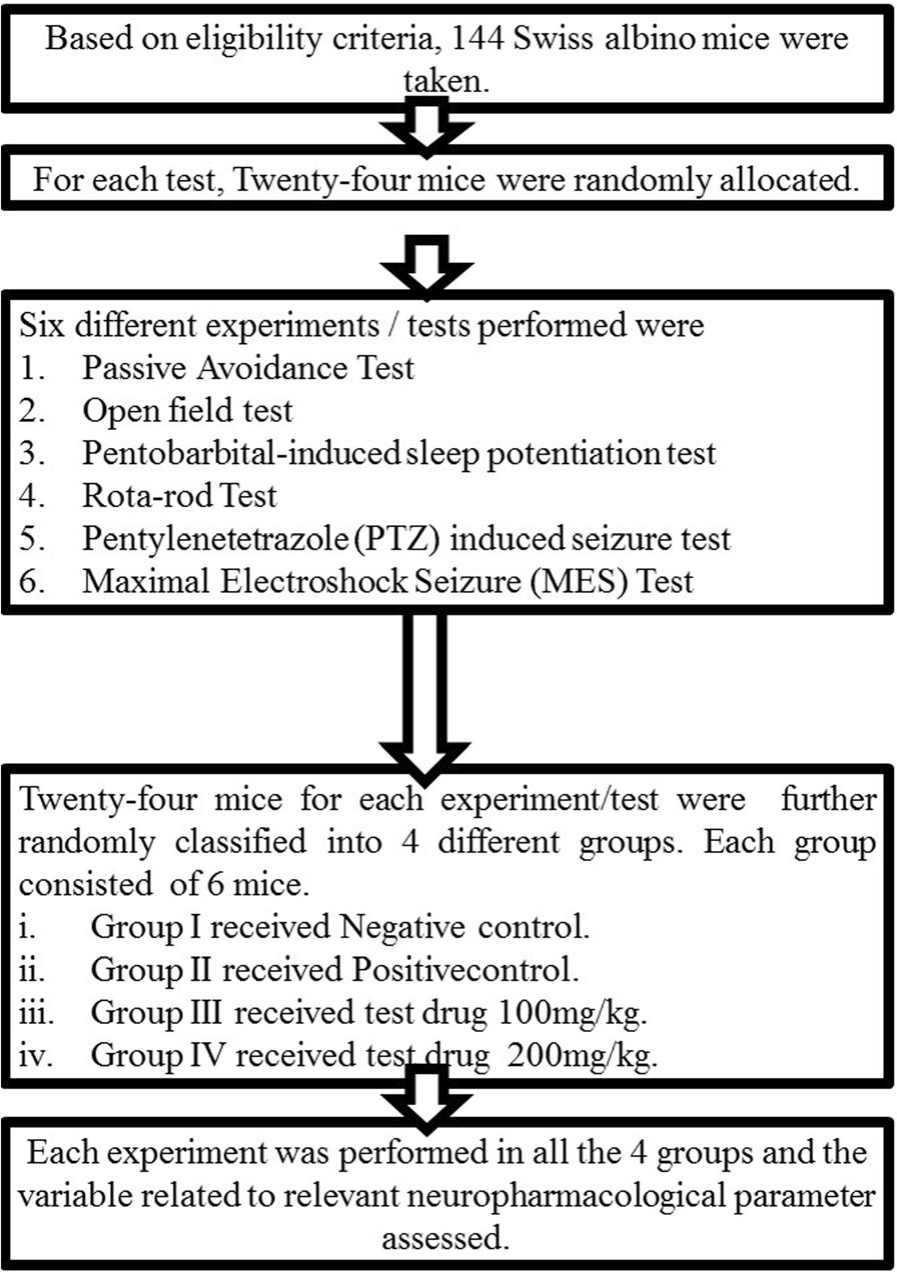
Study Flow Chart

**Table 7:** Animals not showing seizure in MES model

Experimental Group	Animals protected
Negative Control (Normal Saline)	0/6
Positive Control (Phenytoin)	6/6
Test drug 100 mg/kg	0/6
Test drug 200 mg/kg	0/6

**Table 8:** Comparison of Mean duration of tonic hind-limb extension in MES-seizure model among 4 different experimental groups

Experimental Groups	Mean Duration of tonic hind limb extension ± SD (s)
Negative Control (Normal Saline)	13.55 ± 2.75
Positive Control (Phenytoin)	0.00 ± 0.00
Test drug 100 mg/kg	14.58 ± 2.50
Test drug 200 mg/kg	17.57 ± 2.15

All animals in test drug groups (12/12) and Normal Saline group (6/6) exhibited seizure.

All animals (6/6) in phenytoin group were protected against seizure.

Mann–Whitney *U* test (Additional file 1: Table S36) showed that 200 mg/kg group exhibited significantly *(P =* 0.000) longer mean duration of tonic hind-limb extension (17.57 s ± 2.15 s) compared to either to Normal Saline group (13.55 s ± 2.75 s) or to Phenytoin group (00.00s ± 00.00s, i.e., no seizure).

## Discussion

To our knowledge, this is the first report on the detailed neuropharmacological aspects of root extracts of *Ficus benghalensis* L. from Nepal.

Preliminary experiments demonstrated that the high doses (2000 mg/kg) were tolerated without any acute signs of toxicity or mortality. Therefore, one tenth of this dose [37] (i.e. 200 mg/kg) was considered as the highest evaluation dose for pharmacological studies.

### Effect of aqueous extracts of aerial roots of *Ficus benghalensis* L.in learning and memory

In the Passive-avoidance test, we tried to explore the effect of the drug in short-term memory of the mice. Among 3 parameters, step-down latency and step-down error were statistically not significant. Third parameter- Time spent in shock zone pointed towards salutary effects of high dose of the extract in short-term memory of the mice. Central cholinergic receptors are often involved in memory [38]. Therefore, it must have affected the central muscarinic receptor which are involved in Alzheimer brain [39].

Chandra P et al. found that aqueous extract from the bark of *Ficus benghalensis* L. mitigated scopolamine-induced memory deficits in the Passive-avoidance test [11]. Other taxonomically close plants also have proven anti-amnestic effects [40, 41].

### Effect of aqueous extracts of aerial roots of *Ficus benghalensis* L.in anxiety

The open field test is a valid animal model of anxiety-like behavior and is based on the conflict between the exploration of a new environment and the aversion to open spaces from which escape is prevented by a surrounding wall [42].

Animals removed from their acclimatized cage and placed in the open field express fear/anxiety and therefore tend to avoid the aversive centre and spend more time in the protective corners and periphery (thigmotaxis). An increase in central locomotion or in time spent in the central part of the field can be interpreted as an anxiolytic-like effect while the contrary, that is a decrease of these variables, is associated with anxiogenic effects [33].

In our study, mice treated with higher dose of the extract spent more time in the central-square even when compared to Diazepam suggesting anxiolytic effect even better than the conventional axiolytic. To our knowledge, there is no published report regarding effect of *Ficus benghalensis* L.plant parts in anxiety. However, many other plants of same genus do have proven anxiolytic effects like. *Ficus exasperata* Vahl [43] *Ficus religiosa* [44] *Ficus pumila* [45].

Healing anxiety with herbs has been a topic of hot debate in recent years. Natural products are being explored as an alternative for anxiety disorder because of public acceptance of plants and unwanted side-effects of popular anti-anxiety drugs like Benzodiazepines [46].

Several traditional herbal medicines are already known to possess anxiolytic activity Ginseng. Therefore, it most also influence level and activities of several central neurotransmitters noradrenaline, serotonin, gamma-amino butyric acid (GABA), Benzodiazepine (BDZ) affecting anxiety [47].

### Effect of aqueous extracts of aerial roots of *Ficus benghalensis* L.in Pentobartal-induced sleep

We evaluated effect of our test drug in sleep induced by Pentobarbital in mice and compared the duration of resulted sleep with standard sedative drug Benzodiazepine. Taking mean total sleep time as parameter, we could not arrive at a statistically significant effect in sleep.

### Effect of aqueous extracts of aerial roots of *Ficus benghalensis* L.in muscle-co-ordination

Skeletal muscle relaxants are drugs that act peripherally at neuromuscular junction/muscle fibre itself or centrally in the cerebrospinal axis to reduce muscle tone and/or cause paralysis [48]. We tried to evaluate the muscle relaxing property of the mice by documenting how long they could remain in the rotating rod after administering the test drug and compared the fall-off time with that of the standard drug Diazepam. Mice ingesting higher dose of test drug fell from the revolving rod significantly earlier than the standard muscle relaxant Diazepam. To our knowledge there is no published article concerning effect of *Ficus benghalensis* L.on muscle contraction. However, aerial parts of *Ficus caric*a [49], leaves, stem-bark and root-bark of *Ficus sycomorus* [50] and different parts of *Ficus religiosa* [40] do have proven muscle relaxant property.

### Effect of aqueous extracts of aerial roots of *Ficus benghalensis* L.in seizure

Seizure is a paroxysmal event having various manifestations, ranging from dramatic convulsion to an experiential phenomena. It is due to abnormal excessive or synchronous neuronal activity in the brain [51]. Different ion channels (voltage-gated Na^+^ channels, voltage-gated Ca^2+^ channels, voltage-gated K^+^ channels); receptors (excitatory receptors like AMPA (α-amino-3-hydroxy-5-methyl-4-isoxazolepropionic acid) receptors, NMDA (N-methyl-D-aspartate) receptors and inhibitory receptors like GABA receptors and their subtypes, are involved in seizure [47].

Predictive of effectiveness in nonconvulsive (absence or myoclonic) seizures [48], PTZ is known to produce clonic seizure [52] by antagonizing [53] GABA_A_ (Gamma-Aminobutyric Acid) receptors. Convulsion so produced is effectively blocked by Diazepam which increases frequency of opening of GABA_A_ receptor [30].

Electroshock results in a group of neurons firing at an abnormal, excessive, and synchronized manner mimicking tonic phase of Grand mal epilepsy [54, 55]. The tonic phase (especially extensor) is selectively abolished only by drugs effective in generalized tonic–clonic seizures and partial seizure [48, 56].

Our study at low dose significantly delayed PTZ seizure onset. On the other hand, at high dose it increased MES seizure duration. This apparent anomaly in different seizure types is explained by difference in genesis and resulting effective drug in the two seizure type [57]. However, even MES and PTZ models together may not detect all anti-seizure activities [52]. Therefore, few anti-seizure properties of the test drug might have been missed.

Though not in *Ficus benghalensis*, few studies analyzing the effect of other species of same *Ficus* genus have been published which showed mixed results [58, 59].

## Limitations and scope for further study

Though our test-result has brought many things under light, we assume certain shortcomings in our endeavor.

Single acute dose-effect was studied. Sub-acute and chronic dose-effect may not be predicted from these result.

We studied only two most relevant doses of test drug. More doses are needed to establish statistically significant dose-effect relationship.

Distinct climate, soil and environment of the Eastern Nepal might have produced different properties that may reduce the global applicability of our findings.

Individual differences in anatomy, organ function, drug absorption and metabolism among animals are among the myriad of other differences that could have given us different information.

In lack of phytochemical analysis accompanied by cause-effect establishment of individual phyto-constituents, we remain skeptical about extrapolating our report to other plant-parts. Further in-depth research disclosing the phyto-constituents responsible for each neuropharmacological activity along with their correct mode of action is desperately needed.

In spite of all these, we consider our study has high internal and external validity; and our findings can be replicated elsewhere.

## Suggestions

Though technically more demanding, phyto-molecular identification followed by in-vitro receptor-ligand binding studies may be the answer to this dispute.

In any particular effect, the use of antagonists of putative neurotransmitters involved might have shed more lights on the mechanism of the observed effect e.g. In Pentobarbital-induced sleep potentiation test, adding of serotonergic antagonist or anti-glutamate as other positive controls might have cleared the dilemma regarding neurotransmitters concerned.

## Conclusion

Orally administered aqueous extracts of aerial roots of *Ficus benghalensis* L. may have memory-enhancing, anxiolytic, muscle-relaxant and seizure-modifying effect with no neurotoxic effect in Swiss albino mice.

## Additional file

Additional file 1: Table-S1. Step down latency (s). **Table S2.** Mean Step-down latency (s). **Table S3.** One-Way ANOVA Test- mean Step-down latency (s). **Table S4.** Tukey HSD post hoc tests-comparing mean Step-down latency (s). **Table S5.** Step down error. **Table S6.** Comparison of mean Step-down error. **Table S7.** One-Way ANOVA Test- mean Step down error. **Table S8.** Tukey HSD post hoc test comparing mean Step down error. **Table S9.** Time spent in shock zone in s. **Table S10.** Comparison of mean Time spent in shock-zone. **Table S11.** Mann–Whitney *U* test comparing mean time spent in shock-zone (s). **Table S12.** No. of squares crossed. **Table S13.** Comparison of mean Number of squares crossed. **Table S14.** One-Way ANOVA Test- Number of squares crossed. **Table S15.** Tukey HSD post hoc tests-comparing mean Number of squares. **Table S16.** Time spent in Central Square(s). **Table S17.** Comparison of mean Time spent in central-square(s). **Table S18.** Mann–Whitney *U* test comparing mean Time (s) spent in Central Square. **Table S19.** Number of rearing. **Table S20.** Comparison of mean Number of Rearing. **Table S21.** Mann-Whitney *U* test comparing mean number of rearing. **Table S22.** Total sleep time (s). **Table S24.** One-Way ANOVA Test- mean total sleep time in Pentobarbital-induced sleep potentiation test. **Table S25.** Tukey HSD post hoc tests-comparing mean total sleep time. **Table S26.** Fall-off time (s). **Table S27.** Comparison of mean Fall-off time. **Table S28.** Mann–Whitney *U* test comparing mean fall-off time. **Table 29.** Animals not showing seizure. **Table S30.** Latency of seizure onset (s). **Table S31.** Comparison of mean Latency of seizure-onset (s). **Table S32.** Mann–Whitney *U* test comparing mean latency (s) in PTZ-seizure model. **Table S33.** Animals not showing seizure. **Table S34.** Duration of tonic hind limb extension (s). **Table S35.** Comparison of mean duration of tonic hind-limb extension. **Table S36.** Mann–Whitney *U* test comparing mean duration of tonic hind limb extension. (XLSX 11 kb)

## Abbreviations

AMPA: α-amino-3-hydroxy-5-methyl-4-isoxazolepropionic acid
ANOVA: Analysis of variance
BDZ: Benzodiazepine
GABA: Gamma-Aminobutyric acid
HSD: Honest significant difference
IBM: International business machines
IP: Intraperitoneal
MES: Maximal electroshock
NMDA: N-methyl-D-aspartate
PTZ: Pentylenetetrazole
SD: Standard deviation
SPSS: Statistical package for the social sciences
WHO: World Health Organization

## References

[1] Chan K (2003). Some aspects of toxic contaminants in herbal medicines. Chemosphere.

[2] Mahajan MS, Gulecha VS, Khandare RA, Upaganlawar AB, Gangurde HH, Upasani CD (2012). Anti-edematogenic and analgesic activities of Ficus benghalensis. Int J Nutr Pharmacol Neurol Dis.

[3] Henle HV, Chomchalow N. Socio-economic aspects of medicinal and aromatic plant production in Asia. Bangkok: RAPA Publication; 1993.

[4] Kunwar RM, Mahat L, Acharya RP, Bussmann RW. Medicinal plants, traditional medicine, markets and management in far-west Nepal. 2013/04/17 ed. 2013;9:24. 10.1186/1746-4269-9-24 PMC3643841 23587109

[5] Manandhar NP, Manandhar S. Plants and people of Nepal. New york: Timber Press; 2002. Available from: http://www.books.google.com.np/books?id=klAFeYz4YdYC.

[6] Uprety Y, Poudel RC, Shrestha KK, Rajbhandary S, Tiwari NN, Shrestha UB, et al. Diversity of use and local knowledge of wild edible plant resources in Nepal. 2012;8:16 10.1186/1746-4269-8-16 PMC3448512 22546349

[7] Jaiswal R, Ahirwar D (2013). A literature review on ficus bengalensis. Int J Adv Pharmac Res.

[8] Nadkarni KM (1996). Dr. KM Nadkarni’s Indian materia medica: with Ayurvedic, Unani-Tibbi, Siddha, allopathic, homeopathic, naturopathic & home remedies.

[9] Ripu M, Kunwar I, Rainer WB (2006). Ficus (Fig) species in Nepal a review of diversity and indigenous uses. Br Ecol Soc.

[10] Basu NK, Lal SB (1947). Investigations on Indian medicinal plants. Q J Pharm Pharmacol.

[11] Chandra P, Sachan N, Chaudhary A, Yadav M, Kishore K, Ghosh AK (2013). Acute & Sub Chronic Toxicity Studies and Pharmacological Evaluation of Ficus bengalensis L. (Family: Moraceae) on Scopolamine-Induced Memory Impairmentin Experimental Animals. Indian J Drugs.

[12] Deraniyagala SA, Ratnasooriya WD, Perera PS. Effect of aqueous leaf extract of Ficus benghalensis on nociception and sedation in Rats. 2013;11:1–10

[13] Thakare VN, Suralkar AA, Deshpande AD, Naik SR (2010). Stem bark extraction of Ficus bengalensis Linn for anti-inflammatory and analgesic activity in animal models. Indian J Exp Biol.

[14] Taur DJ, Nirmal SA, Patil RY, Kharya MD (2007). Antistress and antiallergic effects of Ficus bengalensis bark in asthma. Nat Prod Res.

[15] Ogunlowo OP, Arimah BD, Adebayo MA. Phytochemical analysis and comparison of in-vitro antimicrobial activities of the leaf, stem bark and root bark of Ficus benghalensis. 2013;3

[16] Gayathri M, Kannabiran K (2009). Antimicrobial activity of Hemidesmus indicus, Ficus bengalensis and Pterocarpus marsupium roxb. Indian J Pharm Sci.

[17] Kaushik NK, Bagavan A, Rahuman AA, Mohanakrishnan D, Kamaraj C, Elango G (2013). Antiplasmodial potential of selected medicinal plants from eastern Ghats of South India. Exp Parasitol.

[18] Govindarajan M, Sivakumar R, Amsath A, Niraimathi S (2011). Mosquito larvicidal properties of Ficus benghalensis L. (Family: Moraceae) against Culex tritaeniorhynchus Giles and Anopheles subpictus Grassi (Diptera: Culicidae). Asian Pacific J Trop Med.

[19] Patil SM, Saini R. Antiulcer Activity of Stem Bark Extract Of Ficus Bengalensis Linn in Rats. Am. J. PharmaTech Res. 2012;2

[20] Pathak K V, Keharia H. Characterization of fungal antagonistic bacilli isolated from aerial roots of banyan (Ficus benghalensis) using intact-cell MALDI-TOF mass spectrometry (ICMS). 2013/02/08 ed. 2013;114:1300–10. 10.1111/jam.12161 23387377

[21] Shukla R, Gupta S, Gambhir JK, Prabhu KM, Murthy PS (2004). Antioxidant effect of aqueous extract of the bark of Ficus bengalensis in hypercholesterolaemic rabbits. J Ethnopharmacol.

[22] Daniel RS, Devi KS, Augusti KT, Sudhakaran Nair CR (2003). Mechanism of action of antiatherogenic and related effects of Ficus bengalensis Linn. flavonoids in experimental animals. Indian J Exp Biol.

[23] Shukla R, Anand K, Prabhu KM, Murthy PS (1994). Hypoglycaemic effect of the water extract of Ficus bengalensis in alloxan recovered, mildly diabetic and severely diabetic rabbits. Int J Diab Develop Count.

[24] Gayathri M, Kannabiran K (2008). Antidiabetic and ameliorative potential of Ficus bengalensis bark extract in streptozotocin induced diabetic rats. Indian J Clin Biochem.

[25] Modak M, Dixit P, Londhe J, Ghaskadbi S, Devasagayam TPA. Recent Advances in Indian Herbal Drug Research Guest Editor : Thomas Paul Asir Devasagayam. 2007;163–73

[26] Manirakiza P, Covaci A, Schepens P (2001). Comparative study on total lipid determination using Soxhlet, Roese-Gottlieb, Bligh & Dyer, and modified Bligh & Dyer extraction methods. J Food Compos Anal.

[27] Sluiter A, Ruiz R, Scarlata C, Sluiter J, Templeton D (2005). Determination of extractives in biomass. Laboratory Analytical.

[28] Kulkarni SK (2010). Handbook of Experimental Pharmacology.

[29] Ghosh MN. Fundamentals of Experimental Pharmacology [Internet]. 5th ed. Hilton & Company: Kolkata; 2011. Available from: http://www.books.google.com.np/books?id=dwn5nAEACAAJ.

[30] Vogel H, Vogel HW, Schölkens BA, Sandow J, Müller G, Vogel WF. Drug Discovery and Evaluation: Pharmacological Assays. 2nd ed. Springer;

[31] Medhi B, Prakash AJ. Practical Manual of Experimental and Clinical Pharmacology [Internet]. 1st ed. Jaypee Brothers, Medical Publishers; 2010. Available from: http://www.books.google.com.np/books?id=CYIHywAACAAJ.

[32] Vohora D, Pal SN, Pillai KK (2000). Effect of locomotor activity on the passive avoidance test for the evaluation of cognitive function. Indian J Pharmacol.

[33] Prut L, Belzung C. The open field as a paradigm to measure the effects of drugs on anxiety-like behaviors: a review. 2003;463:3–33 10.1016/s0014-2999(03)01272-x 12600700

[34] Simon P, Chermat R, Doare L, Bourin M, Farinotti R (1982). Unexpected interactions of some psychotropic drugs with barbital and pentobarbital effects in mice. J Pharmacol.

[35] Singh K, Rauniar G, Sangraula H. Experimental study of neuropharmacological profile of Euphorbia pulcherrima in mice and rats. J Neurosci Rural Pract. 2012;3:311–9. Available from: http://www.ruralneuropractice.com/article.asp?issn=0976-3147;year=2012;volume=3;issue=3;spage=311;epage=319;aulast=Singh. 10.4103/0976-3147.102612 PMC3505323 23188984

[36] Laurence DR, Bacharach AL. Evaluation of drug activities: pharmacometrics [Internet]. Academic Press; 1964. Available from: http://www.books.google.com.np/books?id=rSEcnAEACAAJ

[37] Bhardwaj L, Patil K (2010). Study on efficacy of treatment with Ficus benghalensis leaf extracts on freunds adjuvant induced arthritis in rats. Int J Drug Develop Res.

[38] Brunton L, Chabner B, Knollman B. Goodman and Gilman’s The Pharmacological Basis of Therapeutics [Internet]. 12th ed. USA: Mcgraw-Hill Education; 2010. Available from: http://www.books.google.com.np/books?id=e_yAOpyyaowC

[39] Rinne JO, Rinne JK, Laakso K, Paijarvi RUK (1984). Reduction in muscarinic receptor binding in limbic areas of Alzheimer brain. J Neurol Neurosurg Psychiatry.

[40] Makhija IK, Sharma IP, Khamar D (2010). Phytochemistry and pharmacological properties of Ficus religiosa: an overview. Ann Biol Res.

[41] Kaur H, Singh D, Singh B, Goel RK. Anti-amnesic effect of Ficus religiosa in scopolamine-induced anterograde and retrograde amnesia. Pharm Biol. 2010;48:234–40. Available from: http://www.informahealthcare.com/doi/abs/10.3109/13880200903271306. 10.3109/13880200903271306 20645848

[42] Kopniczky Z, Dochnal R, Macsai M, Pal A, Kiss G, Mihaly A (2006). Alterations of behavior and spatial learning after unilateral entorhinal ablation of rats. Life Sci.

[43] Woode E, Poku RA, Abotsi WKM. Anxiolytic-like effects of a leaf extract of Ficus exasperata Vahl (Moraceae) in Mice. West African Journal of Pharmacy. 2011;22

[44] Ratnasooriya WD, Jayakody J, Dharmasiri MG (1998). An aqueous extract of trunk bark of Ficus religiosa has anxiolytic activity. Med Sci Res.

[45] Ashraf M, V.K, G.Thamotharan, Sengottuvelu S. Evaluation of Anxiolytic Activity of Ficus Pumila L. Leaf-extract in Experimental Animals. International journal of Research in Pharmaceutical and Nano Sciences. 2013;272–82.

[46] Sivaraman D, Muralidharan P, Rahman H, Thoraipakkam C (2012). Ficus hispida Linn in Corticosterone Induced Anxiety in Young Adult Mice. Pharmacologia.

[47] Katzung B, Masters S, Trevor A (2011). Basic and Clinical Pharmacology.

[48] Tripathi KD. Essentials Of Medical Pharmacology [Internet]. 7th ed. New Delhi: Jaypee Brothers Medical Publishers; 2013. Available from: http://books.google.com.np/books?id=anOBygAACAAJ.

[49] Panday DR, Rauniar GP. Effect of root-extracts of Ficus benghalensis(Banyan) in pain in animal models. Journal of Neurosciences in Rural Practice. 2016;7(2):210-215. doi:10.4103/0976-3147.178660. 10.4103/0976-3147.178660 PMC4821927 27114650

[50] Zaku SG, Abdulrahaman FA, Onyeyili PA, Aguzue OC, Thomas SA (2009). Phytochemical constituents and effects of aqueous root-bark extract of Ficus sycomorus L. (Moracaea) on muscular relaxation, anaesthetic and sleeping time on laboratory animals. Afr J Biotechnol.

[51] Longo D, Fauci A, Kasper D, Hauser S, Jameson J, Loscalzo J (2011). Harrison’s Principles of Internal Medicine.

[52] Löscher W. Critical review of current animal models of seizures and epilepsy used in the discovery and development of new antiepileptic drugs. Seizure. 2011;20:359–60. Available from: http://www.sciencedirect.com/science/article/pii/S1059131111000124. 10.1016/j.seizure.2011.01.003 21292505

[53] De Sarro A, Cecchetti V, Fravolini V, Naccari F, Tabarrini O, De Sarro G (1999). Effects of novel 6-desfluoroquinolones and classic quinolones on pentylenetetrazole-induced seizures in mice. Antimicrob Agents Chemother.

[54] Loscher W, Schmidt D (1988). Which animal models should be used in the search for new antiepileptic drugs? A proposal based on experimental and clinical considerations. Epilepsy Res.

[55] McPhee SJ, Lingappa VR, Ganong WF. Pathophysiology of disease: an introduction to clinical medicine [Internet]. USA: Lange Medical Books/McGraw-Hill; 2003. Available from: http://www.books.google.com.np/books?id=IJdrAAAAMAAJ.

[56] Krall RL, Penry JK, White BG, Kupferberg HJ, Swinyard EA (1978). Antiepileptic drug development: II. Anticonvulsant drug screening. Epilepsia.

[57] Bhanushali MM, Makhija DT, Joshi YM (2014). Central nervous system acetonic extract of Ficus carica L. in mice. J Ayurveda Integr Med.

[58] Sandabe UK, Onyeyili PA, Chibuzo GA (2003). Sedative and anticonvulsant effects of aqueous extract of Ficus sycomorus L. (Moraceae) stembark in rats. Veterinarski arhiv.

[59] Singh D, Goel RK (2009). Anticonvulsant effect of Ficus religiosa: role of serotonergic pathways. J Ethnopharmacol.

